# A case report of brain abscess caused by *Nocardia farcinica*

**DOI:** 10.1186/s40001-021-00562-2

**Published:** 2021-08-03

**Authors:** Jiangqin Song, Lian Dong, Yan Ding, Junyang Zhou

**Affiliations:** 1 Laboratory Department, The First People’s Hospital of Tianmen City, Tianmen, 431700 Hubei China; 2 Oncology Department, The First People’s Hospital of Tianmen City, Tianmen, 431700 Hubei China; 3 Hubei Key Laboratory of Embryonic Stem Cell Research, Hubei University of Medicine, Shiyan, 442000 Hubei China; 4 Department of Pathogen Biology and Immunology, Xuzhou Medical University, Xuzhou, 221004 Jiangsu China

**Keywords:** *Nocardia farcinica*, Brain abscess, Case report

## Abstract

**Background:**

Brain abscess due to the *Nocardia* genus is rarely reported and it is usually found in immunocompromised patients. Treatment of *Nocardia* brain abscess is troublesome and requires consideration of the severity of the underlying systemic disease. The difficulties in identifying the bacterium and the frequent delay in initiating adequate therapy often influence the prognosis of patients.

**Case presentation:**

Here, we report a rare case of brain abscess caused by *Nocardia farcinica.* The patient’s medical history was complicated: he was hospitalized several times, but no pathogens were found. At last, bacteria were found in the culture of brain abscess puncture fluid; the colony was identified as *Nocardia farcinica* by mass spectrometry. Targeted antibiotic treatment was implemented, brain abscess tended to alleviate, but the patient eventually developed fungal pneumonia and died of acute respiratory distress syndrome (ARDS).

**Conclusion:**

Brain abscess caused by *Nocardia farcinica* can appear in non-immunocompromised individuals. Early diagnosis, reasonable surgical intervention, and targeted antibiotic treatment are critical for *Nocardia* brain abscess treatment. In the treatment of *Nocardia* brain abscess, attention should paid be to the changes in patients’ immunity and infection with other pathogens, especially fungi, avoided.

## Background

*Nocardia* is an aerobic filamentous environmental Gram-positive bacterium and is usually considered as an opportunistic pathogen, belonging to the order Actinomycetes [1]. *Nocardia* brain abscess is rare and typically found in immunocompromised patients [1]. *Nocardia* infections comprise only 2% of all intracranial abscesses [2], but overall mortality rate can exceed 20% [3, 4]. Brain abscess caused by *Nocardia farcinica* is rarely reported in clinical practice. The treatment is troublesome and the difficulties lie in the timely identification of the bacterium, its inherent resistance to conventional antibiotics and the frequent delay in initiating an effective therapy [5, 6].

Here, we report a patient who suffered from brain abscess caused by *Nocardia farcinica*, although his condition improved after targeted antibiotic treatment, due to basic diseases and long-term use of antibiotics, he developed fungal infection in the lungs and eventually died of ARDS. This study hope to provide experience for the clinical diagnosis and treatment of *Nocardia* brain abscess.

## Case presentation

A male aged 61 years was admitted to our hospital for intermittent fever and cough on August 16, 2019. In the past 4 years, the patient had developed pulmonary infection repeatedly, which improved after anti-infection treatment. He had a history of hypertension, coronary heart disease and bronchiectasis. In the past 1 year, the patient intermittently developed cough, sputum, accompanied by fever, with a body temperature of about 38.0 °C, without afternoon low fever, night sweats and hemoptysis. He was hospitalized for many times, no pathogens were detected during hospital stay. The patient continued to cough and fever intermittently outside the hospital.

**Fig. 1:**
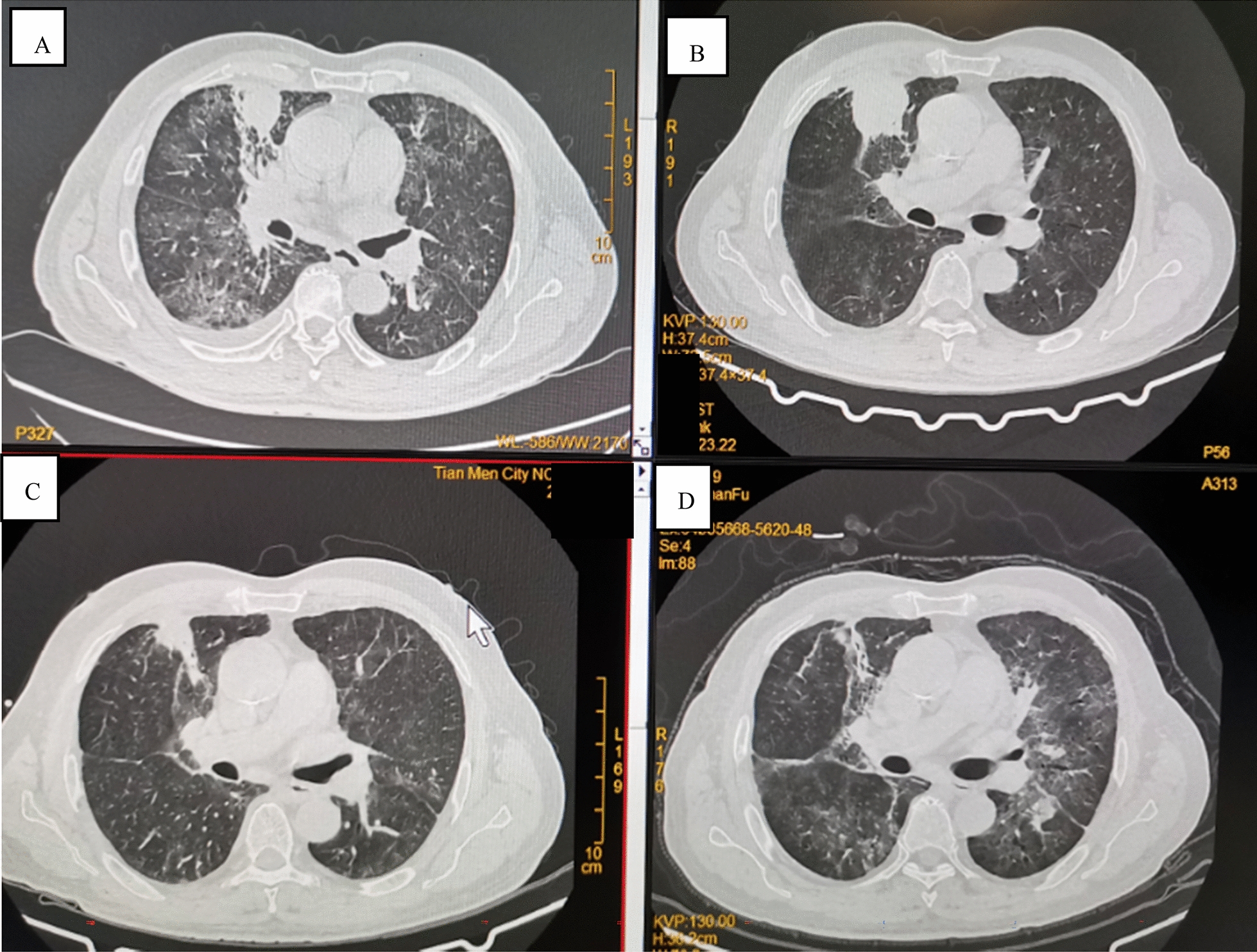
Chest CT images of the patient. **A** Chest CT on June 19. **B** Chest CT August 16. **C** Chest CT on September 14. **D** Chest CT on December 9

On admission, the patient was conscious, with no enlargement of superficial lymph nodes, slightly coarse breathing sounds in both lungs, and a little moist crackles could be heard. On June 19, 2019, chest CT (computed tomography) indicated space occupation in the right upper lung, bilateral lung infective lesion with bronchiectasis, emphysema, bullae of the lung, right pleural effusion (Fig. 1A). On August 16, chest CT indicated that the lesion area of the right upper lung mass was significantly larger than before, accompanied by bronchiectasis, emphysema, and pulmonary bulla (Fig. 1B). After admission, the patient underwent CT-guided percutaneous lung puncture examination, and the tissues were subjected pathological examination and microbial culture. Histopathology showed chronic inflammatory changes accompanied by mild hyperplasia of alveolar epithelium. No bacteria were observed in lung tissue culture. Bronchoscope alveolar lavage fluid (BALF) examination revealed bronchial inflammation. Cytology of lavage fluid exfoliation: no cancer cells detected; mTB-DNA was not detected in BALF by Gene Xpert. No acid-fast bacilli were found in lavage fluid and sputum by acid-fast staining, and no hyphae and spores of bacteria and fungi were found by Gram stain. Mycobacterium culture was negative. IgA, IgG, IgM, C3 and C4 were normal. Blood tests for white blood cells (WBC) 13.6 × 10^9^/L (reference range: 3.5–9.5 × 10^9^/L), C-reactive protein (CRP) 64.67 mg/L (reference range: < 6.0 mg/L), PCT 0.053 mg/L (reference range: 0–0.046 ng/mL).

**Fig 2:**
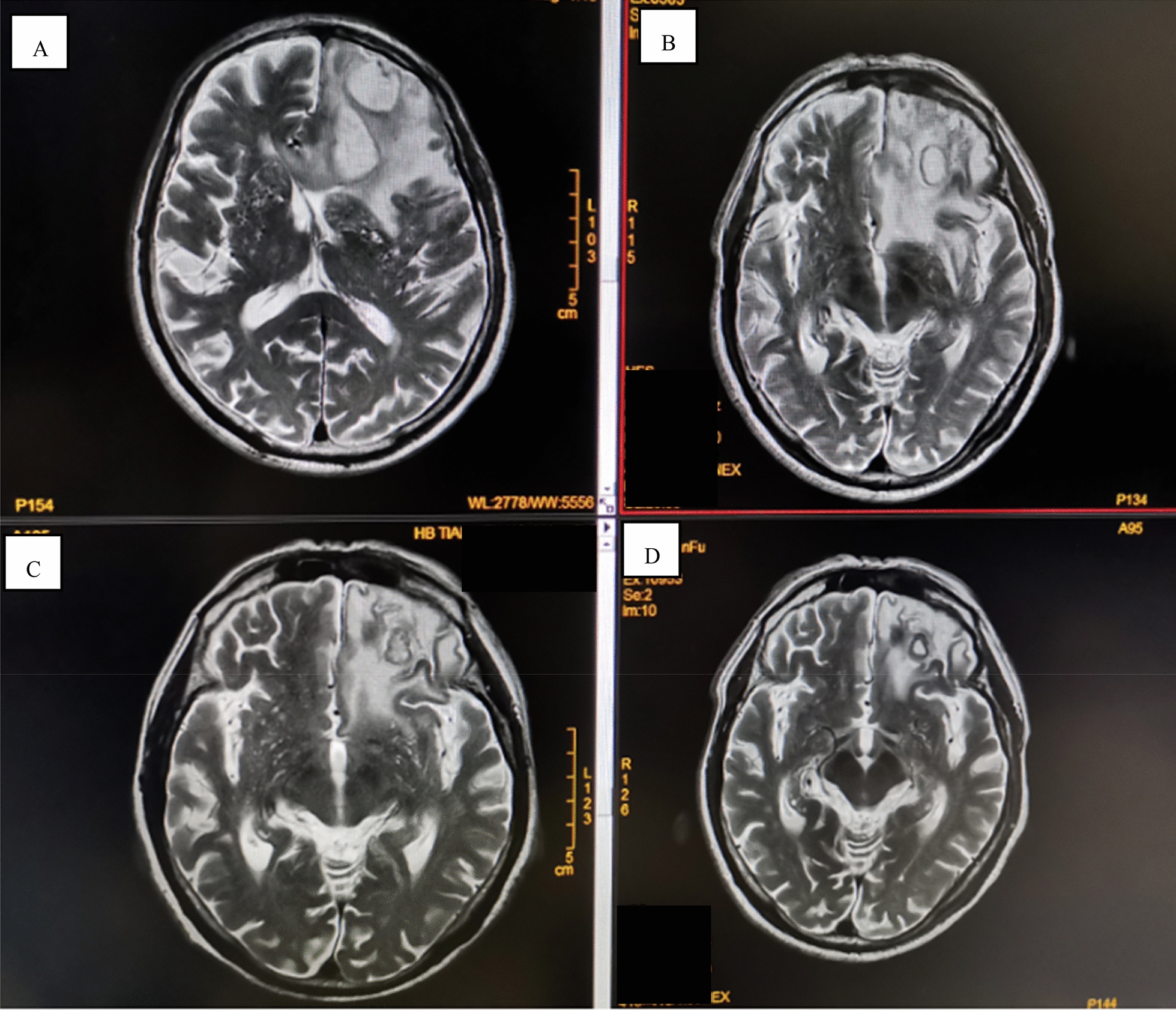
Brain MRI images of the patient. **A** Brain MRI on August 23. **B** Brain MRI September 14. **C** Brain MRI on October 22. **D** Brain MRI on November 22

**Fig. 3:**
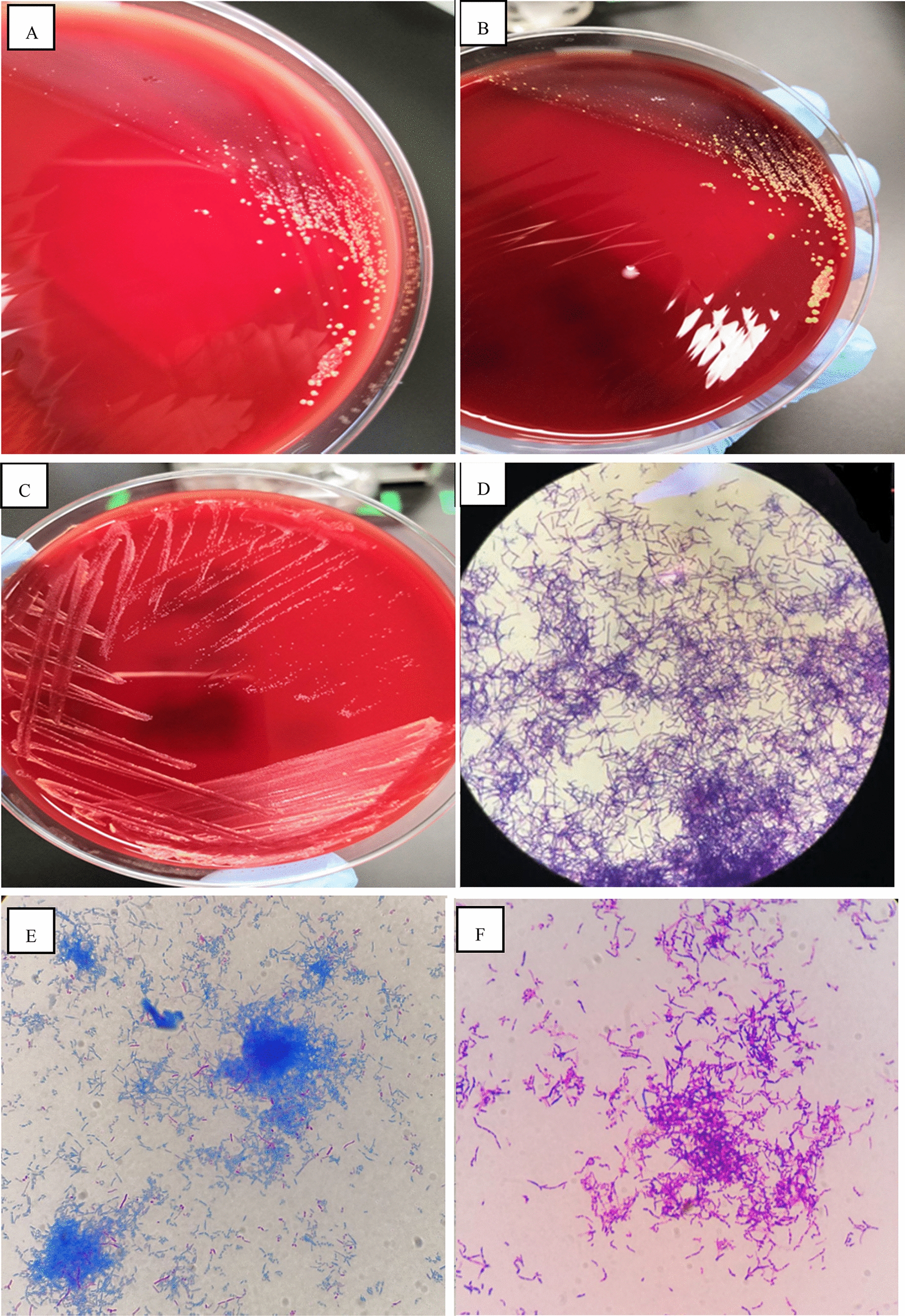
Culture and staining of *Nocardia farcinica.* **A** Images of puncture fluid after 48 h of culture. **B** Images of puncture fluid after 72 h of culture. **C** Images of pure bacteria after 24 h of culture. **D** Gram stain of *Nocardia farcinica.* **E** Acid-fast staining of *Nocardia farcinica.* **F** Weak acid-fast staining of *Nocardia farcinica*

After treatment with cefoperazone sodium + sulbactam sodium, the patient’s symptoms (fever, cough and sputum) improved, but neurological symptoms such as headache, delirium and memory loss appeared on August 16. A magnetic resonance imaging (MRI) scan of the brain suggested space occupation in the left frontal lobe, the maximum cross-sectional area of the lesion was about 35 mm × 52 mm; brain abscess was considered (Fig. 2A). On August 28, the patient underwent minimally invasive puncture drainage under CT-guidance, and about 10 mL yellow purulent fluid was extracted. The puncture fluid was sent to the microorganism laboratory for testing, after 48-h culture, white cotton-like colonies grew (Fig. 3A–C). After smear staining, branching and uneven staining of filamentous bacilli could be seen under the microscope. The mycelia could be wound into clusters to form actinomycetes like particles, Gram stain and the weak acid-fast staining were positive (Fig. 3D–F). The bacteria were identified as *Nocardia farcinic*a by mass spectrometry, with 99.9% credibility. Then, the patient was diagnosed with *Nocardia farcinica* brain abscess. After 16 days of treatment with trimethoprim/sulfamethoxazole (TMP/SMX) (3.0 g, po, bid) and intravenous amikacin (0.4 g, iv, qd), the patient’s temperature returned to normal and his headache completely disappeared, intracranial mass was significantly reduced (Fig. 2B) and the right upper lung mass was significantly absorbed (Fig. 1C). During subsequent treatment, the patient developed nausea and vomiting for many times, which was considered to be caused by cerebral edema. After treatment with mannitol dehydration, the symptoms were relieved. Re-examination of head MRI on October 23 (Fig. 2B) showed that the brain abscess lesions were smaller than before, the brain edema was significantly better than before. The previous anti-infection treatment regimen was continued.

On December 8, the patient had occasional mild chest tiredness, which relieved spontaneously, intermittent cough, nausea and retching, chest CT showed significant increase in lung lesions, partial bronchiectasis, emphysema and bullous lungs appeared (Fig. 1D). The patient developed dyspnea on December 12 accompanied by wheezing sound in both lungs, sputum culture suggested *Candida tropicalis*. The patient was given antifungal and antiasthmatic treatment with itraconazole and doxotheophylline. On December 15, he developed ARDS, blood pressure (BP): 100/75 mmHg, arterial blood oxygen saturation (SaO_2_) 79%. After a series of treatments, including assisted respiration (mask oxygen inhalation), anti-inflammatory (methylprednisolone), antiasthmatic (doxophylline, salbutamol, ipratropium bromide), hyperensort (dopamine), the patient’s dyspnea symptoms were not relieved and blood pressure did not rise (BP 77/52 mmHg). The patient’s family gave up the rescue. Subsequently, the patient went into a deep coma, lost consciousness, and the heart rate dropped. The patient died on the morning of the 16th, and the family refused an autopsy.

## Discussion and conclusion

*Nocardia* is a soil-borne strictly aerobic actinomycete with at least 16 species that can affect human health [7]. *Nocardia* spp. have a predilection for the lungs and brain as foci of infection, particularly in immunocompromised hosts [8]. In this case, the patient was a 61-year-old male with no immunodeficiency disease, but he had bronchiectasis, hypertension, coronary heart disease. Multiple lung infections, 11 hospitalizations, and prolonged antibiotic use in the past 5 years may be the key factors in the patient’s *Nocardia* infection.

*Nocardia farcinica* was the most common species in Nocardia infection, accounting for 24.5% [9]. *Nocardia farcinica* is more prone to affect the central nervous system (CNS) than other species [10, 11]. Clinical manifestations of CNS nocardiosis usually result from local effects of granulomas or abscesses in the brain, less commonly in the spinal cord or meninges [10, 11]. The abscess usually can be identified by CT scan or MRI as a ring enhancement at the capsular phase [12], but needs to be distinguished from tumor, cystic or necrotic foci [13]. In our case, after the patient developed symptom of headache, brain abscess was found by MRI examination. Patient underwent minimally invasive surgery for intracranial abscess puncture and suction under CT-guidance, smear staining and bacterial culture were performed on the drainage fluid, and the cultivated colonies were identified as *Nocardia farcinica* by mass spectrometry.

*Nocardia* identification can be difficult because of the slowly growing pattern of the germ and low positive rate (colonies usually require at least 48 h of incubation although more commonly 3 to 5 days and up to 14 to 21 days), preferably in selective media [14]. To isolate *Nocardia* spp., multiple cerebrospinal fluid (CSF) specimens should be cultured to increase the yield, although it is not uncommon for the bacteria to be recovered only when direct pus is cultured [15]. Certain laboratory techniques like mass spectrometry may help to identify the genus and species. The preferred methods for test of *Nocardia* are 16S rRNA gene analysis and other molecular techniques, such as restriction fragment length polymorphisms and multilocus sequence analysis. Direct abscess drainage seems to be the best method for collection of samples for microbiological confirmation and antibiotic susceptibility testing [16]. *Nocardia* pneumonia often requires bronchoscopy or percutaneous lung biopsy, and a detailed history and thorough physical examination should be taken to adequately assess the presence of spread of the lesions. Cranial CT or MRI should be performed if symptoms or signs suggest intracranial involvement. The patient was considered to have a pulmonary infection caused by inhalation of the bacterium through the respiratory tract and a cerebral abscess caused by haematogenous spread to the brain. In our case, the patient’s repeated sputum culture and BALF tests showed no bacterial growth. Test was negative in the first percutaneous lung biopsy tissue culture, possibly because no valuable lesion tissue was collected at the biopsy site or the use of antibiotics affected the detection rate. This also suggests that *Nocardia* is more difficult to identify than more common bacteria. Bacterial grew after 24 h of culture of puncture fluid, indicating a severe brain infection and suggested that abscess drainage may be good for isolating and culturing *Nocardia.*

Direct smears from surgical samples show Gram-positive, beaded, branching filaments that are partially acid-fast, and thus need to be differentiated from mycobacteria. Colonies usually have a chalky white cotton-like appearance because of the abundant aerial filaments. The smell of moist or wet soil is very characteristic of *Nocardia* spp. colonies [4]. *Nocardia* spp. exhibit variable morphologic appearances depending on the species, the incubation conditions and the duration of incubation. In routine culture media, *Nocardia* spp. appears as bacilli with ramifications and sub-ramifications at right angles that may form coccus in thioglycolate medium after prolonged incubation [7]. The colonies we obtained from the puncture fluid were positive for Gram staining and weak acid resistance for acid-fast staining (Fig. 3D–F). The characteristics of bacterial culture and growth (Fig. 3A–C) are consistent with the above literature reports.

*Nocardia farcinica* brain abscess has a high mortality rate, as high as 20% in immunocompetent patients and 55% in immunocompromised patients. These high rates are attributed to the severity of underlying disease, difficulties in identifying the pathogen, and its inherent resistance to antibiotics, leading to inappropriate or late initiation of therapy [5]. In a study of *Nocardia* isolated from human samples in France, *N. farcinica* was the most frequently isolated species in blood cultures and brain abscesses (21/39, 54% and 19/43, 44.2%, respectively. In the French data, *N. farcinica* was frequently not susceptible to cefotaxime (80% of the isolates), meropenem (73% of isolates) and aminoglycosides (more than 90%) [17]. Taking into account the inherent resistance of *Nocardia farcinica* to third-generation cephalosporins, TMP/SMX’s ability to cross the blood–brain barrier, most authorities recommend TMP/SMX as part of first-line therapy for nocardiosis [18, 19]. Abscesses > 25 mm in diameter and that fail to shrink after 4 weeks of antibiotic therapy should be aspirated to confirm the diagnosis regardless of the immune status of the patient [5]. Empiric treatment of cerebral nocardiosis is well established with the use of parenteral TMP/SMX, amikacin, and imipenem–cilastatin [20, 21]. Recently, extended-spectrum fluoroquinolones such as moxifloxacin have been used successfully against *N. farcinica* cerebral abscess [22]. Because of its ability to cross the blood–brain barrier, TMP/SMX is the treatment of choice and may be effective even when in vitro studies show resistance [20, 23–25]. The abscesses in our patient’s brain was about 35 mm × 52 mm, far more than 25 mm, minimally invasive puncture drainage is of great significance for the identification of pathogenic bacteria and the treatment of patients. After 16 days of treatment with TMP/SMX, the patient’s condition was significantly improved, the lesions in lung and head were also significantly reduced, show a good clinical response (Figs. 1C and 2C). This also suggests that the lung lesions were caused by *Nocardia* *farcinica*.

When the patient was identified with *Nocardia* infection, the lung lesions and brain abscesses were very severe, which affected the patient’s prognosis. After 2 months of continuous antibiotic treatment, the patient suddenly developed dyspnea, an acute outbreak of pulmonary fungal infection (Fig. 1D), laboratory tests identified the pathogen as *Candida tropicalis*, which is also an opportunistic pathogen. The patient developed repeated lung infections, merge a variety of basic diseases, long-term use of many kinds of antibiotics, with low immunity; all these factors may cause patients to have severe fungal infections at the latest stage of treatment. After routine use of antifungal drug, the disease deteriorated and oxygen saturation decreased, eventually resulting in death from ARDS.

In conclusion, brain abscess can be caused by *Nocardia farcinica* in non-immunocompromised individuals and it rarely occurs in clinical. In our case, although the patient’s condition improved after targeted antibiotic treatment (TMP/SMX), due to underlying diseases and long-term use of antibiotics, the central nervous system symptoms appeared lately and delayed diagnosis, the patient eventually died. For pneumonia of unknown cause, a variety of technical means should be used to determine the pathogen as soon as possible: instituting targeted treatment, paying attention to the examination of the brain and other organs. Minimally invasive puncture drainage is of great significance for the diagnosis and treatment of *Nocardia* brain abscess. Because the treatment of *Nocardia* brain abscess requires long-term use of antibiotics, attention should be paid to the changes in patients’ immunity and infection with other pathogens, especially fungi, avoided. Early diagnosis and targeted antibiotic treatment are critical for *Nocardia* brain abscess treatment and prognosis.

## Data Availability

All data are included in this article.

## Abbreviations

ARDS: Acute respiratory distress syndrome
CT: Computed tomography
MRI: Magnetic resonance imaging
BALF: Bronchoscope alveolar lavage fluid
BP: Blood pressure
SaO_2_: Arterial blood oxygen saturation
CNS: Central nervous system
CSF: Multiple cerebrospinal fluid
